# Serine threonine tyrosine kinase 1 is a potential prognostic marker in colorectal cancer

**DOI:** 10.1186/s12885-015-1285-y

**Published:** 2015-04-10

**Authors:** Liang Hu, Hai-Yang Chen, Jian Cai, Yu Zhang, Chen-Ye Qi, Hui Gong, Yan-Xia Zhai, Hao Fu, Guang-Zhen Yang, Chun-Fang Gao

**Affiliations:** 1 Anal-Colorectal Surgery Institute, 150th Hospital of PLA, Luoyang, China; 2 Department of Oncology, 150th Hospital of PLA, Luoyang, China; 3 Department of Colorectal Surgery, 150th Hospital of PLA, Luoyang, China; 4 Department of Clinical Laboratory, 150th Hospital of PLA, Luoyang, China

**Keywords:** STYK1, Colorectal cancer, Survival, Prognosis, Biomarker

## Abstract

**Background:**

Aberrant expression of serine threonine tyrosine kinase 1 (STYK1) has been reported in several human malignancies including colorectal cancer (CRC). However, the prognostic significance of STYK1 expression in CRC remains unknown.

**Methods:**

STYK1 protein expression in paraffin-embedded CRC specimens was determined immunohistochemically. The correlation of STYK1 expression with clinicopathologic features was assessed in a cohort containing 353 patients with primary CRC. Kaplan-Meier and Cox proportional regression analyses were used to evaluate the association between STYK1 expression and patients’ survival.

**Results:**

STYK1 expression was frequently up-regulated in CRC clinical samples at the protein levels and was significantly associated with tumor differentiation grade (p = 0.030), lymph node metastasis (p = 0.004), TNM stage (p = 0.007) and patient death (p < 0.001). Kaplan-Meier analysis indicated that patients with high intratumoral STYK1 expression had a significantly shorter disease-specific survival (DSS) than those with low expression (p < 0.001). Importantly, high levels of STYK1 protein predicted poor DSS for both stage II (p < 0.001) and stage III (p = 0.004) patients. Furthermore, multivariate analyses revealed that STYK1 protein expression was an independent prognostic indicator for both stage II (hazard ratio [HR], 2.472; p = 0.001) and stage III (HR, 2.001; p = 0.004) patients.

**Conclusions:**

Our results suggest that increased STYK1 protein expression correlates with disease progression and metastasis and may serve as a predictor of poor survival in CRC.

**Electronic supplementary material:**

The online version of this article (doi:10.1186/s12885-015-1285-y) contains supplementary material, which is available to authorized users.

## Background

Colorectal cancer (CRC) is the third most common cancer and the fourth most common cancer cause of death globally, accounting for more than 1.2 million new cases and 600,000 deaths every year [1]. Although it was mainly a disease of the developed countries, the incidence of CRC has continued to increase in several regions including East Asia and Central and East Europe during the past decades [2,3]. Currently, the prognosis of CRC patients is highly dependent on clinical stage of the disease [4], however, huge differences of outcome exist even among patients of the same stage category [5]. Therefore, it is urgent needed to search for valuable biomarkers for the early diagnosis and prognosis prediction of patients with CRC.

Receptor protein tyrosine kinases (RPTKs) are important regulators of intracellular signaling pathways mediating diverse cellular and developmental processes and their dysregulation are closely related with the development of cancer [6,7]. Serine threonine tyrosine kinase 1 (STYK1), also known as novel oncogene with kinase domain (NOK), was identified as a new member of the RPTK-like protein family and found to be expressed in several normal human tissues [8,9]. It shares about 30% homology with members of the fibroblast growth factor receptor/platelet-derived growth factor receptor superfamily, and contains a single putative transmembrane domain and a conserved intracellular tyrosine kinase domain, whereas lacks an extracellular domain for binding specific ligands [8]. Therefore, STYK1 may trigger self-phosphorylation and transmit signals downstream without ligand binding. Previous investigations have demonstrated that overexpression of STYK1 could promote cell growth of prostate cancer cells and leukemia cells [10,11], increase growth factor-independent proliferation of BaF3 cells and surface adhesion-independent growth and colony formation of NIH3T3 and BaF3 cells, and induce tumorigenesis and metastasis in nude mice [8,12]. These characteristics indicate that STYK1 has multiple roles in cancer development and progression.

Aberrant expression of STYK1 has been previously documented in human malignancies of the breast, lung, ovary, blood, and prostate [10,11,13-15], and recently, Orang AV *et al.* also reported the significant up-regulation of STYK1 mRNA in CRC tissues [16]. Thus, as an oncogene, STYK1 could be a candidate biomarker for tumor diagnosis and prognosis prediction. It has been showed that STYK1 mRNA might be a tool to support the diagnosis of breast, lung, and colorectal carcinomas [13,14,16]. In addition, the prognostic and therapeutic significance of STYK1 overexpression in human malignant diseases has also been revealed. Chen P *et al.* recently demonstrated that increased expression of STYK1 protein correlates with poor prognosis of patients with non-small cell lung cancer [17]. Nirasawa S *et al.* provided evidence that STYK1 is a novel drug resistance factor and could be a potential predictor of the therapeutic response in acute leukemia [18].

However, the prognostic implication of STYK1 protein expression in CRC has not been investigated. In the present study, we evaluated the phenotypic expression of STYK1 protein immunohistochemically in a large number of CRC clinical samples and examined the correlation of STYK1 expression with clinicopathologic features and with patient survival based on tumor stage. Our data demonstrated that increased expression of STYK1 was closely related to disease progression and metastasis and could serve as an independent predictor of poor prognosis in patients with CRC.

## Methods

### Patients and follow-up

Formalin-fixed paraffin-embedded tissue specimens from 353 stages I–III CRC patients who received curative surgery in 150th Hospital of PLA (Luoyang, China) from July 2006 to December 2009 were retrieved for immunohistochemistry. The study cohort consisted of CRC patients with typical adenocarcinoma histology as confirmed by pathological analysis. Detailed clinicopathologic characteristics of the patients were listed in Table 1. The follow-up period was defined as the interval from the date of surgery to the date of death or last follow-up. The final date of follow-up was 30 September 2014, and the median follow-up time of the cohort was 66 months (range, 2–98 months). Disease-specific survival (DSS) was defined as the interval from the date of surgery to the date that patient died of CRC. Patients alive at the end of follow-up were censored. Patients were excluded from the study cohorts with the following exclusion criteria: previously received any anticancer therapy; impaired heart, lung, liver, or kidney function; previous malignant disease. TNM staging was classified according to the American Joint Committee on Cancer staging manual (seventh edition). Written informed consent was obtained from each patient and this study was approved by the Ethical Committee of 150th Hospital of PLA.

**Table 1:** **Association Between STYK1 Expression and Clinicopathologic Characteristics of CRC Patients in the Study Cohort**

Characteristics		No. of	STYK1 expression		
		patients (%)	Low (%)	High (%)	P value^a^
		(n = 353)	(n = 213)	(n = 140)	
**Age (years)**					0.783
	<60	93 (26.3)	55 (25.8)	38 (27.1)	
	≥60	260 (73.7)	158 (74.2)	102 (72.9)	
**Sex**					0.403
	Female	166 (47.0)	104 (48.8)	62 (44.3)	
	Male	187 (53.0)	109 (51.2)	78 (55.7)	
**Tumor location**					0.908
	Rectum	155 (43.9)	93 (43.7)	62 (44.3)	
	Colon	198 (56.1)	120 (56.3)	78 (55.7)	
**Differentiation grade**					**0.030**
	Well	33 (9.3)	27 (12.7)	6 (4.3)	
	Moderate	256 (72.5)	149 (70.0)	107 (76.4)	
	Poor	64 (18.2)	37 (17.3)	27 (19.3)	
**Tumor size (cm)**					0.950
	<5	152 (43.1)	92 (43.2)	60 (42.9)	
	≥5	201 (56.9)	121 (56.8)	80 (57.1)	
**Local invasion**					0.055
	T_1_-T_2_	48 (13.6)	35 (16.4)	13 (9.3)	
	T_3_-T_4_	305 (86.4)	178 (83.6)	127 (90.7)	
**Lymph node metastasis**					**0.004**
	N_0_	209 (59.2)	140 (65.7)	69 (49.3)	
	N_1_	108 (30.6)	58 (27.2)	50 (35.7)	
	N_2_	36 (10.2)	15 (7.1)	21 (15.0)	
**TNM stage**					**0.007**
	I	43 (12.2)	31 (14.6)	12 (8.6)	
	II	166 (47.0)	109 (51.2)	57 (40.7)	
	III	144 (40.8)	73 (34.2)	71 (50.7)	
**Death**					**<0.001**
	No	211 (59.8)	150 (70.4)	61 (43.6)	
	Yes	142 (40.2)	63 (29.6)	79 (56.4)	

^**a**^ Pearson chi-square test or Fisher exact test was used for comparison between subgroups. Bold type indicates statistical significance.

### Tissue microarray and immunohistochemistry

Tissue microarrays (TMAs) containing the specimens from 150th Hospital of PLA were constructed (Shanghai Biochip Company Ltd, Shanghai, China). Immunohistochemistry of paraffin-embedded tissue sections was performed as described previously [19]. Briefly, sections were deparaffinized and rehydrated. The endogenous peroxidase activity was blocked with 3% H_2_O_2_ for 10 minutes. Antigens were retrieved with citrate buffer (10 mM, pH 6.0) for 15 minutes at 100°C in a microwave oven. After blocking, the sections were incubated with a primary anti-STYK1 antibody (Abcam, ab97451) with 1:200 dilution at 4°C overnight in a moist chamber followed by incubated with an anti-rabbit peroxidase-conjugated secondary antibody (Santa Cruz) at room temperature for 30 minutes. Finally, the visualization signal was developed with diaminobenzidine (Dako) and the slides were counterstained with hematoxylin.

Stained sections were evaluated in a blinded manner without prior knowledge of the clinical data using the German immunoreactive score (IRS), as described previously [20,21]. Briefly, staining intensity was graded as “0” (negative), “1” (weak), “2” (moderate) and “3” (strong); staining extent was graded as “0” (<5%), “1” (5-25%), “2” (25-50%), “3” (50-75%) and “4” (>75%). Values of the staining intensity and the staining extent were multiplied as a final IRS of STYK1 expression, which ranged from 0 to 12. The median value of the IRS was chosen as the cut-off for high and low STYK1 expression levels based on a measure of heterogeneity according to the log-rank test with respect to DSS, as described previously [22]. An IRS of ≥ 6 was used to define tumors with high STYK1 expression and an IRS of < 6 was used to indicate tumors with low STYK1 expression. If there was a discrepancy in individual evaluations, then the cases were reevaluated together with other pathologists to reach a consensus.

### Statistical analysis

Pearson chi-square test or Fisher exact test was used to analyze the relationship between STYK1 expression and clinical features. Kaplan-Meier analysis with log-rank test was used to compare patients’ survival between subgroups. The effect of each variable on survival was determined by the Cox multivariate regression analysis. All statistical analyses were carried out using SPSS PASW Statistics 18.0 software (SPSS, Inc., Chicago, IL), and *p* value < 0.05 were considered to be statistically significant.

## Results

### Expression of STYK1 protein in primary CRC tissues

**Figure 1:**
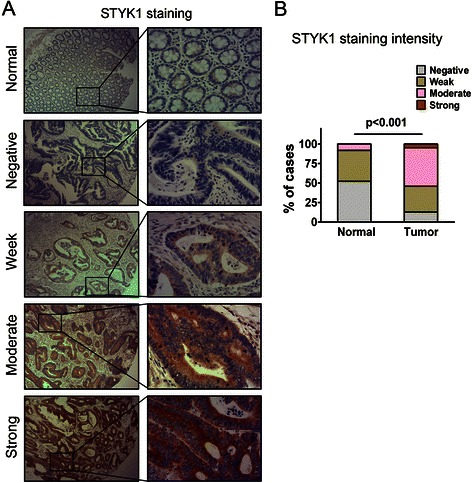
**Expression of STYK1 protein in primary CRC tissues. (A)** Immunohistochemical characteristics of STYK1 in cancerous and adjacent normal mucosa specimens. Representative patterns of STYK1 expression were shown. (Magnification, left panel, ×40; right panel, ×200) **(B)** Percentage of cases with different staining intensity of STYK1 in the tumor or adjacent normal tissues in the study cohort. (p < 0.001).

To determine the phenotypic expression of STYK1 protein in CRC clinical samples, immunohistochemical analysis was performed using a tissue microarray containing 353 pairs of CRC specimens. Each pair consisted of cancerous and adjacent normal colorectal mucosa specimens derived from the same patient. As representatively shown in Figure 1A, positive staining of STYK1 was observed mainly in the cytoplasm of cancer cells and the immunostaining intensity of STYK1 protein was graded to four levels including negative, weak, moderate and strong. In all, 4.6% (16/353) of the cancerous specimens showed strong staining, 50.1% (177/353) of the cases showed moderate staining, 33.1% (117/353) of the cases showed weak staining and only 12.2% (43/353) of the cases showed negative staining of STYK1 protein. In striking contrast, 51.6% (182/353) of the corresponding adjacent normal tissues showed negative staining, 40.2% (142/353) of the cases showed weak staining, 8.2% (29/353) of the cases showed moderate staining and none of the cases showed strong staining of STYK1 (Figure 1B, p < 0.001). Thus, STYK1 protein expression was frequently upregulated in CRC.

### Relationship between STYK1 protein expression and clinical features

The clinicopathologic characteristics of the 353 CRC patients were summarized in Table 1. To evaluate the association between STYK1 protein levels and clinicopathologic characteristics, the patients were classified into high and low intratumoral STYK1 expression subgroups with the median IRS value as the cut-off. As the results shown in Table 1, high expression of STYK1 protein was significantly associated with poor tumor differentiation (p = 0.030), increased lymph node metastasis (p = 0.004), advanced TNM stage of the disease (p = 0.007) and increased death (p < 0.001). While, there were no significant associations between STYK1 expression and patient age (p = 0.783), sex (p = 0.403), tumor location (p = 0.908), tumor size (p = 0.950) or local invasion (p = 0.055).

### Prognostic values of STYK1 expression for patients with CRC

**Figure 2:**
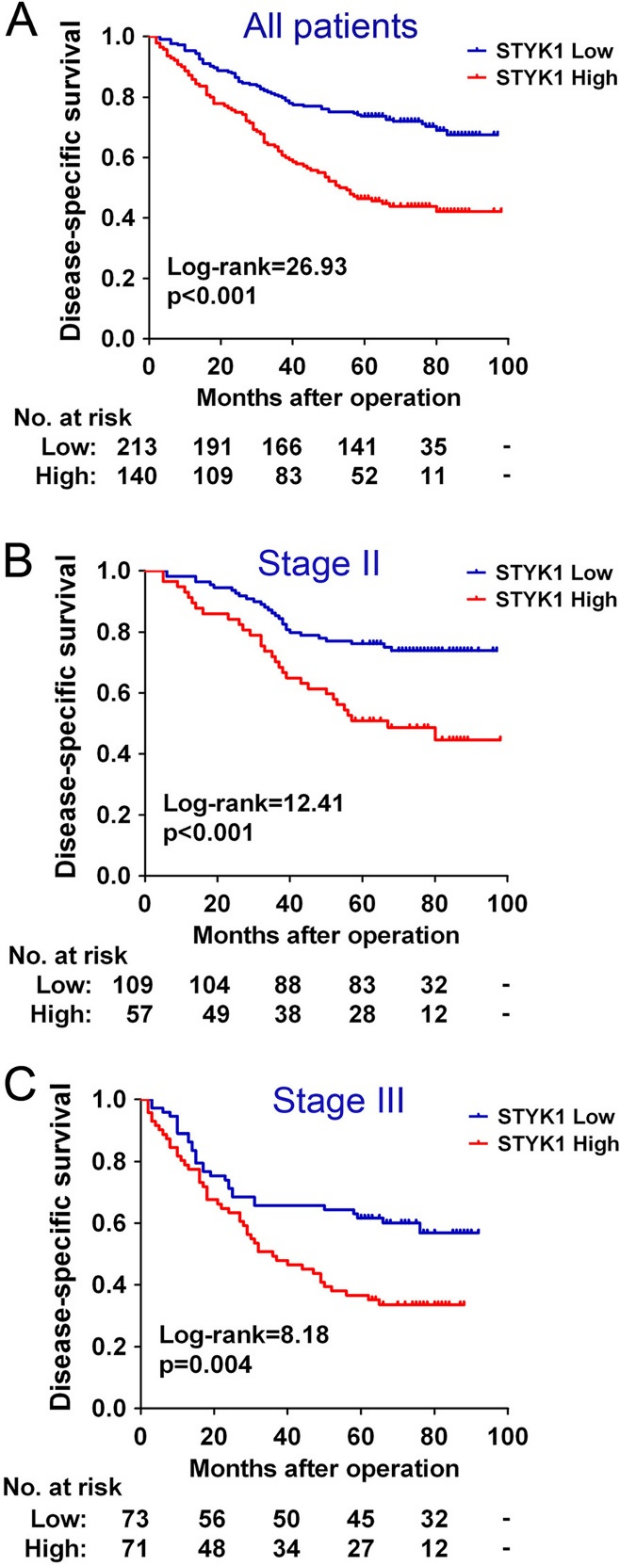
**Kaplan-Meier survival analysis for CRC patients.** Kaplan-Meier curves for disease-specific survival of all CRC patients in the study cohort **(A)**, stage II patients **(B)** and stage III patients **(C)** according to STYK1 expression status. Patients were divided into high and low STYK1 expression subgroups with the median IRS value as the cut-off. The p-value was determined using the log-rank test.

To investigate the relationship between STYK1 expression and the clinical outcome of CRC patients, Kaplan-Meier analysis and the log-rank test were used to assess the effects of STYK1 on patient survival. The results showed that patients with high intratumoral STYK1 expression had a significantly shorter DSS than those with low STYK1 expression (Figure 2A, p < 0.001). For patients with low STYK1 expression, the 1-year, 3-year and 5-year DSS were 95.3%, 80.3% and 73.7%, while patients with high STYK1 expression had 1-year, 3-year and 5-year DSS of 85.7%, 62.1% and 46.4%, respectively. In our cohort, patients who had advanced TNM stage (stage III) tumors had a significantly worse prognosis compared with those who had early stage (stage I and II) tumors (Additional file 1, p < 0.001).

To test the prognostic value of STYK1 in patients with the same stage category, we stratified patients based on stage and further performed survival analysis according to the expression of STYK1 protein. Notably, high expression of STYK1 protein significantly predicted poor DSS not only in stage II patients (Figure 2B, p < 0.001) but also in stage III patients (Figure 2C, p = 0.004).

To assess whether STYK1 expression represents an independent prognostic indicator in CRC, the effect of each variable on survival was determined by the Cox regression analysis. Univariate analyses revealed that differentiation grade (HR = 1.495, 95%CI = 1.004-2.227, p = 0.048), TNM stage (HR = 2.262, 95%CI = 1.625-3.150, p < 0.001) and STYK1 expression (HR = 2.340, 95%CI = 1.679-3.262, p < 0.001) showed significantly higher hazard ratios for a poor prognosis. The parameters that significantly correlated with survival in the univariate analysis were further assessed by multivariate analysis. The results of the multivariate analysis revealed that, besides TNM stage (HR = 2.033, 95%CI = 1.455-2.840, p < 0.001), STYK1 expression was an independent prognostic indicator (HR = 2.116, 95%CI = 1.513-2.960, p < 0.001) (Table 2).

**Table 2:** **Univariate and Multivariate Analyses of STYK1 Expression and Disease-Specific Survival of Patients in the Study Cohort**

Variables	Categories	Univariate analysis			Multivariate analysis^b^		
		HR	95% CI	P value^c^	HR	95% CI	P value^c^
Age ( years)	≥60 / <60	1.182	0.801-1.745	0.399			
Sex	Male / female	1.223	0.878-1.704	0.234			
Tumor location	Colon / rectum	1.042	0.745-1.456	0.811			
Tumor size (cm)	≥5 / <5	1.266	0.904-1.773	0.170			
Differentiation grade	Poor / well + moderate	1.495	1.004-2.227	**0.048**			
TNM stage	III / I+ II	2.262	1.625-3.150	**<0.001**	2.033	1.455-2.840	**<0.001**
STYK1 expression^**a**^	High / low	2.340	1.679-3.262	**<0.001**	2.116	1.513-2.960	**<0.001**

*Abbreviations*: HR, hazard ratio; 95% CI, 95% confidence interval.

^**a**^ For STYK1, the median value of the IRS was used as the cut-off point for definition of subgroups (low expression and high expression groups).

^**b**^ Multivariate models were adjusted for age, sex, tumor location, tumor size, differentiation grade, and TNM stage.

^**c**^ Bold type indicates statistical significance.

In addition, the independent prognostic significance of STYK1 protein expression on CRC-specific survival based on TNM stage was further evaluated with a Cox regression model. The results showed that increased expression of STYK1 protein was an independent indicator of a poor prognosis for both stage II patients (HR = 2.472, 95%CI = 1.445-4.229, p = 0.001) and stage III patients (HR = 2.001, 95%CI = 1.251-3.200, p = 0.004). For stage III patients, differentiation grade also was significantly associated with patient survival (HR = 1.680; 95%CI = 1.033-2.733, p = 0.037) (Table 3).

**Table 3:** **Multivariate Analyses of STYK1 Expression and Disease-Specific Survival for Stage II and III Patients in the Study Cohort**

Variables	Categories	Stage II Patients			Stage III Patients		
		HR	95% CI	P value^b^	HR	95% CI	P value^b^
Age ( years)	≥60 / <60	1.421	0.705-2.865	0.326	1.475	0.883-2.463	0.138
Sex	Male / female	0.956	0.556-1.645	0.872	1.132	0.718-1.784	0.594
Tumor location	Colon / rectum	0.819	0.472-1.420	0.478	1.158	0.729-1.840	0.534
Tumor size (cm)	≥5 / <5	1.324	0.768-2.284	0.312	1.389	0.863-2.235	0.176
Differentiation grade	Poor / well + moderate	0.854	0.325-2.245	0.749	1.680	1.033-2.733	**0.037**
STYK1 expression^**a**^	High / low	2.472	1.445-4.229	**0.001**	2.001	1.251-3.200	**0.004**

*Abbreviations*: HR, hazard ratio; 95% CI, 95% confidence interval.

^**a**^ For STYK1, the median value of the IRS was used as the cut-off point for definition of subgroups (low expression and high expression groups).

^**b**^ Bold type indicates statistical significance.

## Discussion

RPTKs have been implicated in the regulation of a variety of cellular processes including cell proliferation, survival, differentiation, migration and apoptosis through various signaling pathways [6,7]. According to previous reports, humans express at least 58 RPTKs [23]. Normally, the activity of RPTKs is highly controlled. While, perturbation of RPTKs by aberrant expression or mutation could promote cellular transformation and tumorigenesis [24]. Numerous studies have implicated RPTKs as oncogenes, and several RPTKs such as EGFR, VEGFR, HER2 have been selected as targets in cancer therapy [25]. Phylogenetic tree analysis indicated that STYK1 belongs to a distant member of FGFR/PDGFR family [8]. Functional studies demonstrated that STYK1 has the ability to promote cell proliferation, induce transformation and tumorigenesis and facilitate tumor cell invasion and metastatic progression [8,10-12]. Mechanistic investigations revealed that STYK1 could concomitantly activate both the MAPK and PI3K pathways, indicating that it may be standing out as a distinct member of the RPTK family [8]. In addition, a recent study showed that STYK1 interacts and forms complexes with both Akt and GSK-3beta and enhances phosphorylation of GSK-3beta at its Ser9 residue via Akt phosphorylation at Thr308 [26]. Given the importance of the MAPK, PI3K, Akt and GSK-3beta signalings in the pathogenesis of human cancers, these molecular findings may, in part, account for the oncogenic property of STYK1 protein.

Due to the potential significance of STYK1 in cancer biology, its clinical relevance in human malignancies has aroused increasing attention. The diagnostic implication of STYK1 mRNA has been demonstrated in cancers of breast, lung and colorectum [13,14,16]. In addition, the prognostic value of STYK1 protein expression in non-small cell lung cancer has also been revealed [17]. Moreover, it has been clarified recently that measurement of STYK1 mRNA expression could be a potential marker for predicting the therapeutic outcome of various types of acute leukemia [18]. To date, only one study investigated the expression of STYK1 in CRC. Using 36 pairs of CRC tissues, Orang AV *et al.* performed qRT-PCR analysis and found that STYK1 mRNA expression was significantly elevated in cancerous tissues when compared to matched adjacent non-cancerous counterparts [16]. Nevertheless, the prognostic significance of STYK1 expression has not been assessed in CRC.

To the best of our knowledge, the current study is the first to report the prognostic value of STYK1 protein expression in primary CRC tissues. Immunohistochemical analysis of 353 paired CRC specimens revealed that STYK1 protein was mainly localized in the cytoplasm and 87.8% (310/353) of the cancerous tissues tested were STYK1 positive staining, whereas only 48.4% (171/353) of the adjacent normal mucosa tissues showed weak-moderate STYK1 immunoreactivity. These results were in agreement with the previous findings of Orang AV *et al*. at the mRNA level [16] and definitely confirmed the significant up-regulation of STYK1 protein in CRC. Similar to what we observed in the present study, earlier investigations reported that STYK1 was overexpressed in several solid and hematological tumors [10,11,13-15,17]. However, the underlying mechanism of STYK1 overexpression in CRC is currently unknown and requires further investigation.

According to our results, increased expression of STYK1 protein was significantly correlated with poor tumor differentiation, increased lymph node metastasis and advanced TNM stage of the disease, indicating that STYK1 may be involved in the progression of CRC. Likewise, Chen P *et al.* also demonstrated a significant correlation of STYK1 protein expression with the grade of tumor differentiation, TNM stage and lymphatic metastasis in non-small cell lung cancer [17]. However, Orang AV *et al.* reported that the high expression of STYK1 mRNA in CRC was only correlated with the increased tumor size in their cohort [16]. Similar to Orang AV’s findings, an earlier study conducted by Amachika T *et al.* showed that there were no obvious correlations between STYK1 mRNA expression and clinicopathologic features of patients with lung cancer [14]. Of note, Orang AV *et al.* and Amachika T *et al.* studied the mRNA expression levels of STYK1 in fresh-frozen samples, while Chen P *et al.* and we investigated STYK1 protein levels in formalin-fixed paraffin-embedded tissue specimens. Therefore, these discrepancies may be due to the different type of studies and samples.

Interestingly, in the present study, we observed a significant association between increased STYK1 protein expression and poor patient survival in both univariate and multivariate survival analyses. Our findings are also similar to the results from Chen P *et al.* [17], in that they demonstrated that increased expression of STYK1 protein was significantly associated with shortened survival in patients with lung cancer. In addition, our results demonstrated that TNM stage also is an important prognostic factor in CRC, which is consistent with the well established adverse prognostic effect of tumor stage [27] and confirm that our cohort was representative and that the survival analyses were valid. More importantly, our stage-based survival analyses confirmed that increased expression of STYK1 protein not only significantly predicted poor DSS but also was an independent predictor of poor prognosis in stage II as well as in stage III patients. These findings should be of particular interest especially for stage II patients. It is well known that CRC prognosis is highly stage dependent and that TNM staging is still a solid basis for therapeutic decision-making, however, dilemmas still exist with regard to the selection of stage II patients for appropriate treatment. In general, stage II CRC patients have a more favorable outcome than stage III patients [1]. Nevertheless, a subgroup of stage II patients have an increased risk of early recurrence and death. Therefore, identification of this high-risk subgroup of stage II CRC patients by markers is of great clinical need for prognosis prediction. Thus, our current results suggest that STYK1 protein expression status could be a promising biomarker to stratify stage II patients into distinct risk subgroup and guide individualized therapy choices.

The potential mechanism for the prognostic importance of STYK1 oncogene overexpression in CRC is unknown and needs to be further investigated. Nevertheless, the reported abilities of STYK1 to increase cell proliferation, induce malignant transformation, and promote tumorigenesis and metastasis may, at least partly, explain the reason why increased STYK1 protein expression correlates with disease progression and poor prognosis of patients with CRC.

There were several shortcomings in this work. Although our results demonstrated prognostic values for STYK1 protein expression in a cohort of CRC patients, they did not elucidate the role of STYK1 expression in CRC development. In addition, due to the limitation of follow-up period, the median survival time of patients with low STYK1 expression had not been reached, thus, results from the present work could not accurately reflect the survival of patients in this subgroup. Moreover, disease relapse monitoring was incomplete for 84 of the 353 patients, resulting in the loss of disease-free survival data, which is also important for a biomarker validation. Further studies are needed to confirm our findings before clinical translation and to provide a better understanding of the function and mechanism of STYK1 expression in the development and progression of CRC.

## Conclusions

We report here, for the first time, that upregulated expression of STYK1 protein was significantly correlated with disease progression and poor postoperative prognosis of CRC patients. Thus, as a novel RPTK, STYK1 might represent a promising prognostic biomarker and potential therapeutic target for patients with CRC.

## Additional file

Additional file 1: **Kaplan-Meier curves for****disease-specific****survival of all CRC patients according to TNM stage of the disease.** The p-value was determined using the log-rank test.

## Abbreviations

CRC: Colorectal cancer
STYK1: Serine threonine tyrosine kinase 1
DSS: Disease-specific survival
IHC: Immunohistochemistry
IRS: Immunoreactive score
RPTK: Receptor protein tyrosine kinase
MAPK: Mitogen-activated protein kinase
PI3K: Phosphoinositide 3-kinase
GSK: Glycogen synthase kinase
Akt: v-akt murine thymoma viraloncogene homolog
CI: Confidence interval
HR: Hazard ratio
