# Supplementary figures and images for: Serine threonine tyrosine kinase 1 is a potential prognostic marker in colorectal cancer
Source: BMC Cancer. 2015 Apr 10;15:246. doi: 10.1186/s12885-015-1285-y (PMC4404069; doi:10.1186/s12885-015-1285-y)

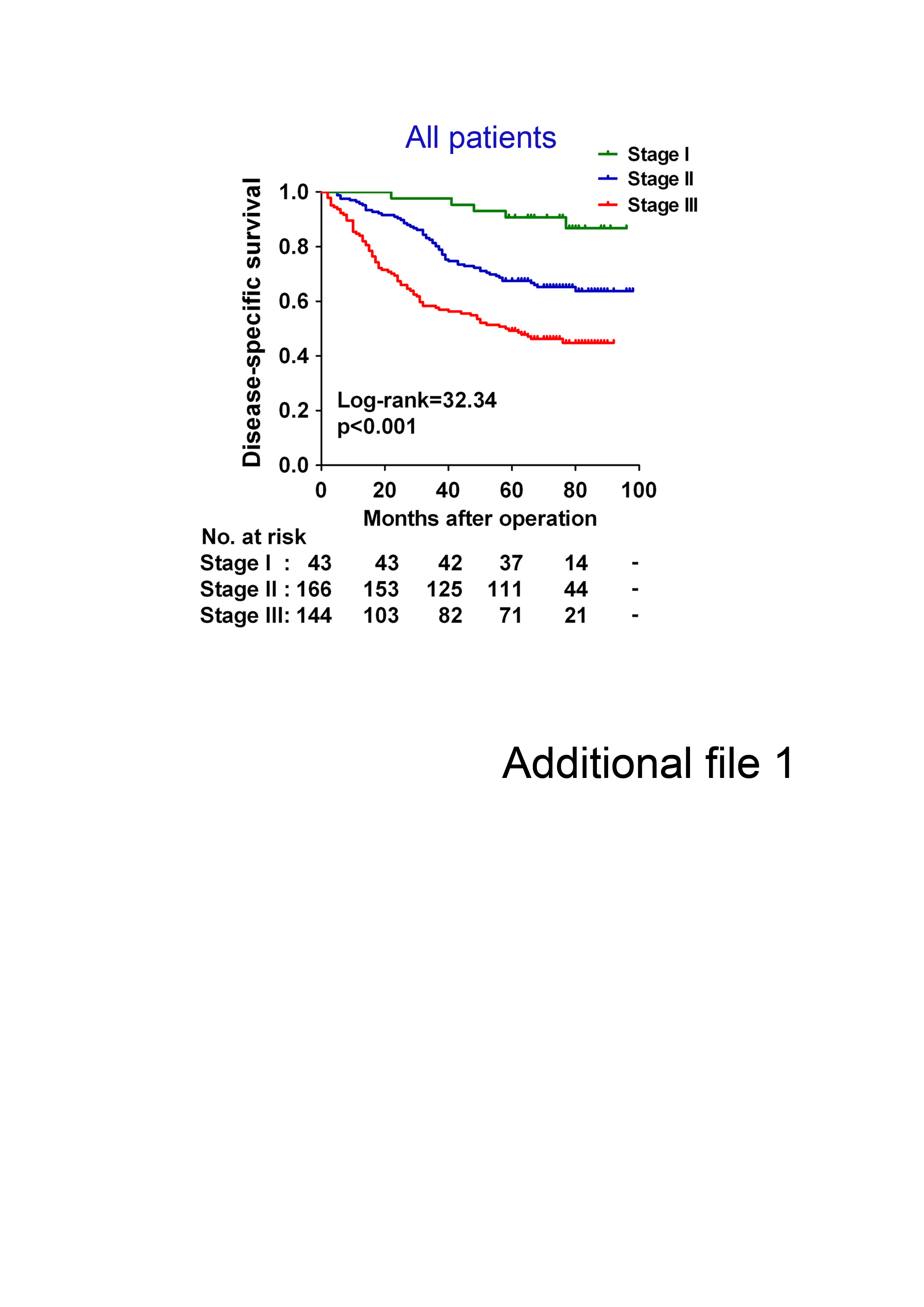

Supplement: Additional file 1: — Kaplan-Meier curves fordisease-specificsurvival of all CRC patients according to TNM stage of the disease. The p-value was determined using the log-rank test. [file 12885_2015_1285_MOESM1_ESM.jpeg]
